# Immunologic Factors Associated with Differential Response to Neoadjuvant Chemoimmunotherapy in Triple-Negative Breast Cancer

**DOI:** 10.3390/jpm14050481

**Published:** 2024-04-30

**Authors:** Robert J. Seager, Heidi Ko, Sarabjot Pabla, Maria-Fernanda Senosain, Pawel Kalinski, Erik Van Roey, Shuang Gao, Kyle C. Strickland, Rebecca Ann Previs, Mary K. Nesline, Stephanie Hastings, Shengle Zhang, Jeffrey M. Conroy, Taylor J. Jensen, Marcia Eisenberg, Brian Caveney, Eric A. Severson, Shakti Ramkissoon, Shipra Gandhi

**Affiliations:** 1 Labcorp Oncology, Buffalo, NY 14263, USA; pablas@labcorp.com (S.P.); senosam@labcorp.com (M.-F.S.); vanroee@labcorp.com (E.V.R.); gaos@labcorp.com (S.G.); zhangs7@labcorp.com (S.Z.); conroj2@labcorp.com (J.M.C.); 2 Labcorp Oncology, Durham, NC 27710, USA; heidi.ko@labcorp.com (H.K.); kyle.strickland@labcorp.com (K.C.S.); rebeccaann.previs@labcorp.com (R.A.P.); neslim1@labcorp.com (M.K.N.); stephanie.hastings@labcorp.com (S.H.); jenset2@labcorp.com (T.J.J.); eric.severson@labcorp.com (E.A.S.); shakti.ramkissoon@labcorp.com (S.R.); 3 Roswell Park Comprehensive Cancer Center, Buffalo, NY 14263, USA; pawel.kalinski@roswellpark.org; 4 Department of Pathology, Duke University Medical Center, Duke Cancer Institute, Durham, NC 27710, USA; 5 Department of Obstetrics & Gynecology, Duke University Medical Center, Duke Cancer Institute, Division of Gynecologic Oncology, Durham, NC 27710, USA; 6 Labcorp, Burlington, NC 27710, USA; eisenbm@labcorp.com (M.E.); caveneb@labcorp.com (B.C.); 7 Wake Forest Comprehensive Cancer Center and Department of Pathology, Wake Forest School of Medicine, Winston-Salem, NC 27710, USA

**Keywords:** breast cancer, triple-negative, tumor microenvironment, chemotherapy, immunotherapy, immune checkpoint inhibitors, neoadjuvant, combination therapy, gene expression profiling, immune profiling

## Abstract

**Background:** KEYNOTE-522 resulted in FDA approval of the immune checkpoint inhibitor pembrolizumab in combination with neoadjuvant chemotherapy for patients with early-stage, high-risk, triple-negative breast cancer (TNBC). Unfortunately, pembrolizumab is associated with several immune-related adverse events (irAEs). We aimed to identify potential tumor microenvironment (TME) biomarkers which could predict patients who may attain pathological complete response (pCR) with chemotherapy alone and be spared the use of anti-PD-1 immunotherapy. **Methods:** Comprehensive immune profiling, including RNA-seq gene expression assessment of 395 immune genes, was performed on matched FFPE tumor samples from 22 stage I-III TNBC patients (14 patients treated with neoadjuvant chemotherapy alone (NAC) and 8 treated with neoadjuvant chemotherapy combined with pembrolizumab (NAC+I)). **Results:** Differential gene expression analysis revealed that in the NAC group, IL12B and IL13 were both significantly associated with pCR. In the NAC+I group, LCK and TP63 were significantly associated with pCR. Patients in both treatment groups exhibiting pCR tended to have greater tumor inflammation than non-pCR patients. In the NAC+I group, patients with pCR tended to have greater cell proliferation and higher PD-L1 expression, while in the NAC group, patients with pCR tended to have lower cancer testis antigen expression. Additionally, the NAC+I group trended toward a lower relative dose intensity averaged across all chemotherapy drugs, suggesting that more dose reductions or treatment delays occurred in the NAC+I group than the NAC group. **Conclusions**: A comprehensive understanding of immunologic factors could potentially predict pCR to chemotherapy alone, enabling the avoidance of the unnecessary treatment of these patients with checkpoint inhibitors.

## 1. Introduction

KEYNOTE-522, a phase III randomized clinical trial, evaluated the addition of pembrolizumab, an immune checkpoint inhibitor (CPI), to an intensive anthracycline- and carboplatin-containing chemotherapy regimen in patients with early-stage high-risk triple-negative breast cancer (TNBC). This study showed that the addition of pembrolizumab to chemotherapy in the neoadjuvant setting led to a remarkable improvement in pathologic complete response (pCR) from 30 to 50% with chemotherapy alone to 65%. Given these promising results, the U.S. FDA approved pembrolizumab in combination with chemotherapy in the neoadjuvant setting for patients with early-stage and locally advanced TNBC [1]. However, there is a subset of patients who may not benefit from the addition of pembrolizumab to chemotherapy, and the use of pembrolizumab can lead to debilitating adverse effects and significant financial toxicity. In the KEYNOTE-522 study, the addition of pembrolizumab was associated with 13% grade 3–5 immune-related adverse events (irAEs), some of which can be life-threatening and debilitating, including cardiomyositis, encephalitis, and adrenal insufficiency, among others, versus only 1% in the group with NAC alone [1].

Currently, due to the lack of biomarker selection for trial eligibility, all patients with high-risk stage II and III TNBC receive pembrolizumab in the neoadjuvant setting, resulting in the potential overtreatment of a large majority of patients. Hence, there is an unmet need to identify biomarkers which could identify patients who may attain pCR with chemotherapy alone and be spared the side effects of the combination with pembrolizumab [2,3,4,5,6,7,8,9].

## 2. Methods

### 2.1. Patients and Clinical Data

The primary analysis cohort was compiled from age- and stage-matched formalin-fixed paraffin-embedded (FFPE) pre-treatment tumor samples from 22 stage I-III TNBC patients, collected under BDR 162722. There was sufficient pre-treatment tissue available for 8 patients treated with neoadjuvant chemotherapy (NAC) combined with pembrolizumab (NAC+I). Of the 8 patients that received neoadjuvant pembrolizumab, 6 were matched 1:2 to non-pembrolizumab controls and 2 were matched 1:1. Of the 1:2-matched patients, a stage I pembrolizumab-treated patient was matched to a stage II control patient of a similar age and a stage III pembrolizumab-treated patient was matched to a stage II control patient of similar age, as there were no other suitable controls available. Pathological responses were documented as pathological complete response (pCR: ypT0/Tis ypN0 (absence of invasive cancer in the breast and axillary lymph nodes)) vs. non-pCR based on pathology reports.

### 2.2. Quality Assessment of Clinical FFPE Tissue Specimens

FFPE tissue blocks were cut into 5 µm sections and placed onto positively charged slides. A section from each sample was hematoxylin-and-eosin-stained and tumor quality was assessed by a board-certified anatomical pathologist, including tumor content, necrosis, and tissue preservation quality. Any samples with less than 5% tumor tissue or greater than 50% necrosis were not analyzed. Tissue from 3 to 5 unstained slides was required to achieve the RNA requirement for the assay (10 ng), with or without tumor macrodissection.

### 2.3. Nucleic acid Isolation and Quantitation

After acoustics-based coextraction of DNA and RNA using the truXTRAC FFPE extraction kit (Covaris, Inc., Woburn, MA, USA), RNA from each sample was quantified using ribogreen staining on a Qubit^®^ fluorometer (Thermo Fisher Scientific, Waltham, MA, USA).

### 2.4. Genomic and Immune Profiling

RNA sequencing was used to measure gene expression, assessing 395 transcripts on all samples that met previously validated quality control thresholds for mapped reads, on-target reads, mean read length, mean depth, uniformity, and percentage of valid reads [3,4]. Libraries from extracted RNA were prepared and sequenced to an appropriate depth on Ion Torrent^®^ S5XL (Thermo Fisher Scientific, Waltham, MA, USA) sequencers.

### 2.5. Data Analyses

Data from RNA sequencing were processed using the immuneResponseRNA plugin for Torrent Suite (Thermo Fisher Scientific, Waltham, MA, USA), which generated absolute read counts for each transcript [10]. For each gene, absolute read counts were converted to a percentile rank (0–100) compared against a reference population of 735 solid tumors of 35 histological subtypes [10]. Genomic profiling was performed via the Illumina Trusight^®^ Oncology 500 (TSO500) analysis pipeline (v2.1.0.60; Illumina, San Diego, CA, USA) [11].

Further data analysis was conducted using R software (v4.3.0). Two published gene expression signatures with demonstrated associations with immunotherapy response were calculated for each sample: the cell proliferation signature (CP, poor/moderate/high) [12,13] and the tumor immunogenic signature (TIGS, weak/moderate/strong) [14]. The cancer testis antigen burden (CTAB) biomarker was calculated by summing the gene expression ranks of 17 CTAs (*BAGE*, *CTAG1B* (*NY-ESO-1*), *CTAG2* (*LAGE-1A*), *GAGE1*, *GAGE10*, *GAGE12J*, *GAGE13*, *GAGE2*, *MAGEA1*, *MAGEA10*, *MAGEA12*, *MAGEA3*, *MAGEA4*, *MAGEC2*, *MLANA*, *SSX2*, and *XAGE1B*), resulting in an integer value between 0 and 1700 for each sample.

Continuous variables were compared between patient groups using the Wilcoxon Rank-Sum test. Kaplan–Meier (KM) analysis was used for survival analysis of overall and recurrence-free survival data. Treatment response was compared between patient groups using Fisher’s exact test without continuity correction. Differential gene expression analyses were performed using a negative binomial distribution Wald test. In all cases, *p*-values less than 0.05 were considered to be significant.

## 3. Results

### 3.1. Cohort Characteristics and Clinical Outcomes

There was no difference in the distribution of baseline demographic, treatment, or pathological characteristics between the patients treated with NAC vs. NAC+I. The only exception was a difference in PD-L1 expression observed by RNA sequencing (Table 1). The pCR rate for the entire cohort was 72.7% (16/22), with no statistically significant difference in pCR rate between the two treatment groups. A significant difference was also not observed in the overall or recurrence-free survival between the two treatment groups.

### 3.2. Tumor Microenvironmental Biomarkers and pCR

**Figure 1:**
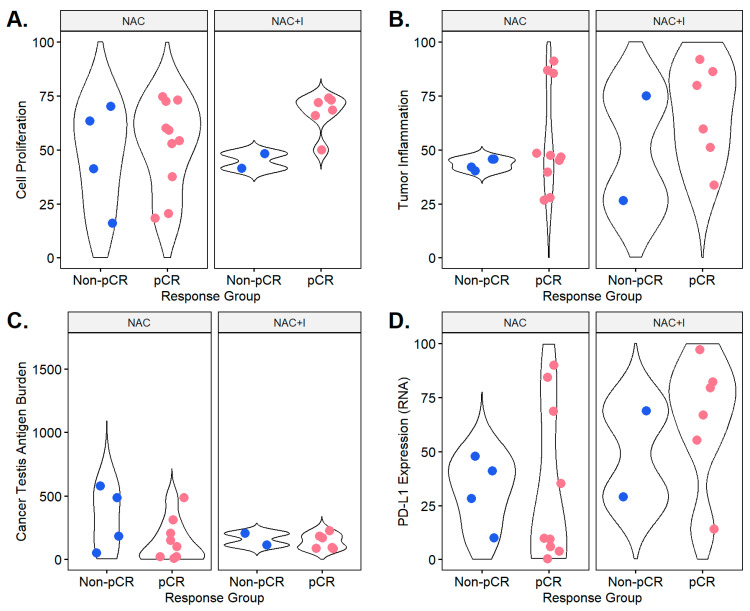
Distributions of cell proliferation (**A**), tumor inflammation (**B**), cancer testis antigen burden (**C**), and PD-L1 expression measured by RNA-seq (**D**) for groups treated with neoadjuvant chemotherapy alone (NAC) and neoadjuvant chemotherapy combined with immunotherapy (NAC+I). Individual biomarker values for pCR and non-pCR patients are denoted by overlaid colored points.

Comparing the distributions of four gene expression-derived microenvironmental biomarkers between the pCR and non-pCR groups within each treatment group, some key trends emerge. First, patients who achieved pCR in the NAC+I group had greater cell proliferation than those without pCR (Figure 1A). Second, in both treatment groups, patients with high tumor inflammation achieved pCR (Figure 1B). Third, patients who achieved pCR in the NAC group had lower CTA expression than non-pCR patients (Figure 1C). Finally, patients with pCR in the NAC+I group had higher than average PD-L1 expression, while pCR patients in the NAC group had higher or lower than average PD-L1 expression (Figure 1D).

### 3.3. Gene Expression and pCR

**Figure 2:**
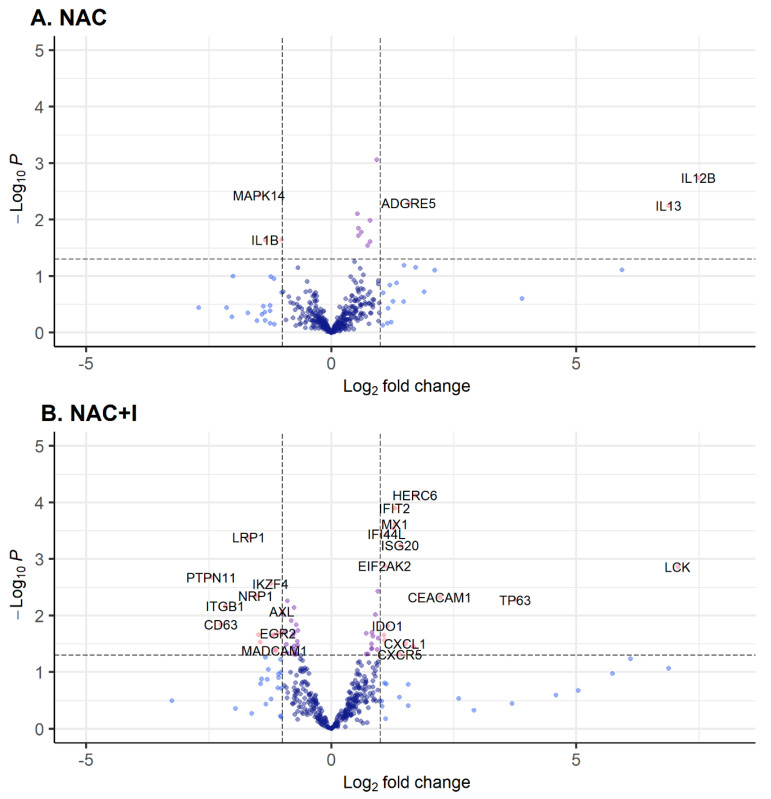
Volcano plots showing gene expression differences between pCR and non-pCR groups in the (**A**) neoadjuvant chemotherapy alone (NAC) and (**B**) neoadjuvant chemotherapy combined with immunotherapy (NAC+I) treatment groups. Significantly differentially expressed genes with a fold change greater than 2 are shown in red. Those associated with pCR are to the right of the central vertical line and those associated with non-pCR are to the left of the central vertical line.

Differential gene expression analysis comparing patients achieving pCR to those who did not, revealed unique gene expression profiles associated with both pCR and non-pCR in the NAC (Figure 2A) and NAC+I (Figure 2B) subgroups. Interestingly, there was no overlap between the lists of genes associated with pCR and non-pCR across both treatment groups (Table 2). In each treatment group, a small number of genes were particularly strongly associated with pCR. In the NAC treatment group, *IL12B* and *IL13* were both significantly associated with pCR and had a gene expression fold change of greater than 10 between pCR and non-pCR groups. In the NAC+I group, *LCK* and *TP63* were similarly significantly associated with pCR and had a gene expression fold change of greater than 10 between pCR and non-pCR groups.

### 3.4. Therapy Dose and pCR

**Figure 3:**
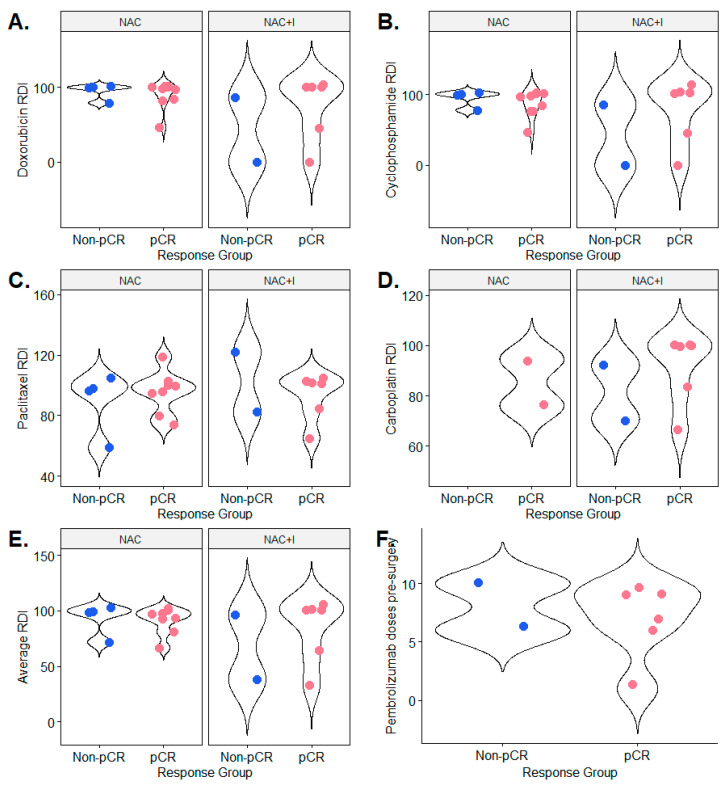
Chemotherapy dose and pCR in both treatment groups. Violin plots of relative dose intensity (RDI) in the pathological complete response (pCR) and non-pCR groups in each treatment group, neoadjuvant chemotherapy alone (NAC) and neoadjuvant chemotherapy in combination with pembrolizumab immunotherapy (NAC+I), for four chemotherapy drugs: doxorubicin (**A**), cyclophosphamide (**B**), paclitaxel (**C**), carboplatin (**D**), and the average of all drugs used to treat each patient (**E**). Also shown are violin plots of the distributions of the number of doses of pembrolizumab administered to patients in the pCR and non-pCR groups within the NAC+I treatment group (**F**).

Recognizing the limitations imposed by the small cohort size, we observed two notable, though non-significant, trends in the treatment dose and response data. Doxorubicin (Figure 3A) and cyclophosphamide both trended toward lower relative dose intensity (RDI) in the NAC+I group compared to the NAC group. While this trend is not observable for paclitaxel (Figure 3C) or carboplatin (Figure 3D), the average RDI for all drugs appears to follow this trend as well (Figure 3E). Based on this, we can posit that there may have been more treatment dose reductions or interruptions in the NAC+I than in the NAC group.

## 4. Discussion

Our study, in contrast to KEYNOTE-522 [1], did not show a difference in pCR among those treated with NAC or NAC+I. These findings are similar to findings from the NeoTRIP [15] and GeparNuevo studies [16]. NeoTRIP enrolled and randomized patients to receive neoadjuvant carboplatin and nab-paclitaxel with or without atezolizumab. In the GeparNuevo study, patients with early-stage, locally advanced TNBC were randomized to receive neoadjuvant nab-paclitaxel followed by doxorubicin-cyclophosphamide with or without durvalumab. In both these studies, no difference in pCR was observed in the two arms. This raises the question as to whether the use of neoadjuvant immune checkpoint inhibition would add more benefit to patients with a unique tumor biomarker profile.

We found that in the NAC+I treatment group, tumors exhibiting pCR trended toward having greater cell proliferation (CP) than non-pCR patients. Previous studies have demonstrated that increased cell proliferation in the tumor microenvironment can result from tumor growth or immune activation, and a balance between tumor and immune proliferative activity, demonstrated by a moderate CP score, is a predictor of improved response to immunotherapy [14]. Our study findings are similar to the NeoTRIP study, where fractions of proliferating CD8^+^TCF1^+^T cells and MHCII^+^ cancer cells were dominant predictors of response. In addition, this study also showed that cancer-immune interactions with B cells and granzyme B^+^ T cells were also predictors of response to immunotherapy [17].

We found that patients exhibiting pCR trended toward having higher tumor inflammation (as assessed by TIGS score), among tumors treated with both NAC and NAC+I. These findings are similar to NeoTRIP, where higher stromal TILs ≥ 40% was associated with a higher pCR rate in both the atezolizumab and the chemotherapy arms. Interestingly, the NeoPACT clinical trial [9,18] further investigated the immune gene score (14-gene IGG signature) to identify patients who would derive added benefit from neoadjuvant immune checkpoint inhibition. However, when the same gene signature was investigated in patients treated with the same chemotherapy backbone of carboplatin and docetaxel in the NeoSTOP (without immunotherapy) trial, this improvement in pCR was not observed. Similar findings were observed in another real-world study where high TILs were associated with higher pCR with chemoimmunotherapy [19].

In our study, the patients in the NAC group exhibiting pCR also trended toward having lower CTA expression than non-pCR patients. In the absence of therapies leveraging the inherently immunogenic nature of these antigens, this relationship between CTA expression and adverse clinical outcomes is well documented [20]. The expression of these antigens by tumor cells is associated with increased proliferation and immune evasion and, thus, tumor growth development [21,22]. This result suggests that increased attention to markers of immunogenicity and immune escape beyond PD-L1 may allow for the identification of additional patients who might benefit from immunotherapy.

Our study also showed that among the NAC+I group, tumors exhibiting pCR trended toward having higher PD-L1 expression, while in the NAC group, this was not observed. These findings are similar to the ones of the NeoTRIP study [15], where higher PD-L1 expression was the most significant factor influencing the rate of pCR (OR = 2.08, *p* < 0.0001) in the immunotherapy arm. This study shows that higher PD-L1 may help select patients who would preferentially benefit from neoadjuvant immunotherapy and chemotherapy combination as opposed to neoadjuvant chemotherapy alone, as also shown in a meta-analysis [23]. However, PD-L1 did not seem to differentiate tumors that attained pCR vs. non-pCR in the KEYNOTE 522 trial.

Taken together, these descriptors of the tumor-immune microenvironment in both NAC and NAC+I treatment contexts suggest a web of relationships between the tumor cells and other microenvironmental actors, like the immune system, best described by multiple biomarkers [24]. In particular, these results suggest that the presence of inflammation alone does not necessitate immunotherapy, as it has a role in the response to neoadjuvant chemotherapy alone. The inclusion of other biomarkers, such as PD-L1 and CTA expression, in treatment selection decisions should be further explored in order to identify patients who will derive the most benefit from immunotherapy while weighing the risks of toxicity.

Our analysis of treatment dose and response across the NAC and NAC+I groups revealed that, on average, chemotherapy drugs prescribed to patients across both treatment groups trended toward lower relative dose intensity (RDI) in the NAC+I group compared to the NAC group. This suggests that there may have been more treatment reductions in the NAC+I group than in the NAC group. One of the distinguishing factors between the two matched treatment groups is the use of immunotherapy, a possible driving factor of chemotherapy dose reductions due to the added toxicity profile. These results suggest that the biomarker-based selection of patients likely to derive the most benefit from neoadjuvant immunotherapy could help reduce potential debilitating immunologic adverse effects by curtailing unnecessary immunotherapy for patients outside this group.

Differential gene expression analysis comparing pCR to non-pCR patients within both treatment groups revealed four mutually exclusive lists of genes, each associated with a clinical outcome in each treatment group. The mutual exclusivity of the lists of genes differentially expressed in pCR and non-pCR across both treatment groups suggests the primary engagement of distinct mechanisms in the response to both treatment protocols, despite chemotherapy being a component of both. The genes most strongly associated with pCR (FC > 10, *p* < 0.05) in each treatment group also suggested possible functional underpinnings of the observed response to both treatments.

*IL12B* was strongly associated with pCR in the NAC treatment group. *IL12B* encodes the p40 subunit of the dimeric cytokines IL-12 and IL-23 [25]. It is also intimately involved in both the innate immune response and in subsequent T-cell polarization [26]. In particular, it is a key component in both the differentiation of T helper type 1 (Th1) cells and the maintenance of Th17 cells through IL-12 and IL-23 signaling, respectively [27,28,29]. Notably, though not in breast cancer, the expression of *IL12* has been associated with an inhibition of tumor cell growth after treatment [30].

*IL13* was also strongly associated with pCR in the NAC treatment group. IL13 is another cytokine associated with a wide array of immunoregulatory functions [31]. It is involved in alternative (M2) macrophage activation induced by the T helper cell 2 (Th2) cytokines IL-4 and IL-13, rather than the classical (M1) Th1-induced macrophage activation pathway mediated by interferon gamma (IFN-γ) [32,33]. It is also a promoter of B-cell proliferation in a similar manner to IL-4 [34,35]. However, unlike IL-4, IL-13 does not have an apparent role in CD4 T-cell differentiation into Th-2 cells, but appears to be more important as a regulator of the effector aspect of the inflammation response [36].

*LCK* was strongly associated with pCR in the NAC+I treatment group. LCK is an Src-family kinase that regulates T-cell functions, including the initiation of TCR signaling, T-cell development, and T-cell homeostasis [37]. LCK has been shown to affect T-cell differentiation decision-making between CD4 and CD8 T cells [38], and in naïve T cells, it is intimately involved in the regulation of TCR activity [39,40]. This central role of LCK in T-cell regulation has made it a target of research in improving the efficacy of immunotherapy and the durability of the resulting immune response [37].

*TP63* was also strongly associated with pCR in the NAC+I treatment group. TP63 is a member of the TP53 family, though it has important distinctions from the more well-known TP53 tumor suppressor gene [41]. In contrast to TP53, TP63 has a role in epithelial morphogenesis and adult epithelial stem cell maintenance and differentiation [42,43,44,45]. However, it is also known to be widely involved in tumorigenesis and cancer progression, including the inhibition of metastasis [45,46,47]. Consequently, the expression of particular TP63 isoforms has been connected with clinical outcomes across multiple cancer subtypes, and there is a potential tumor suppressive role for TP63 that is more prominent for certain isoforms [48,49].

Although the small sample size did not allow our study to demonstrate statistically significant differences in certain studied aspects, we believe that these results highlight the importance of a collective, multi-faceted interrogation of multiple aspects of tumor microenvironments when making treatment decisions for patients. Taken together, some notable patterns emerge. First, the two genes most strongly associated with response to chemotherapy alone were both interleukins, suggesting a key role of immune activity and inflammation in the response to chemotherapy. Second, both genes associated with T-cell activation and response, like *LCK*, and genes involved in other aspects of tissue organization and tumor biology, like *TP63*, have roles in immunotherapy response. Third, the association of immunomodulatory signaling proteins with pCR in both treatment groups suggests that tumor inflammation is essential for inducing durable responses to both cytotoxic chemotherapy and immunotherapy. Use of immune checkpoint inhibitors is associated with the development of significant irAEs. Our recent clinical trial indicated the feasibility of enhancing the TME immunologic signature using novel systemic approaches, raising the question of whether the responses to neoadjuvant chemotherapy can be enhanced using additional low-toxicity approaches [50]. As above, there remains an unmet need to identify biomarkers to identify subsets of patients who could benefit from immunotherapy. 

## 5. Conclusions

There was no difference in pCR rates between the NAC and NAC+I groups in our study. There was a trend toward higher pCR with NAC+I in tumors with higher cell proliferation and PD-L1 positivity, while lower cancer testis antigen expression was associated with a trend towards higher pCR in the NAC group. Importantly, there was a trend towards higher pCR in both the NAC and NAC+I groups with higher tumor inflammation. Our study highlights that future real-world studies are essential to evaluate the prognostic value of the immune, cell proliferation, and cancer testis antigen signatures employed in this study. More than simply restating the oncobiological adage that no two tumors are the same, these results suggest that, even in our small cohort, microenvironmental diversity presented by patients can result in different treatment outcomes, even for patients selected for particular therapies by traditional criteria. A multi-marker approach would significantly aid clinicians in deciding which patients are most likely to benefit from the addition of neoadjuvant immunotherapy to chemotherapy, sparing a subset of patients who may not benefit from the combination treatment due to immunologic toxicities. Thus, for these reasons, the development of predictive biomarkers of treatment response and their combined use to classify tumors based on the interplay between multiple microenvironmental factors is essential for the successful integration of immunotherapy with chemotherapy as a combination treatment strategy.

## Figures and Tables

**Table 1:** Cohort characteristics.

Characteristics		Treatment Group			NAC+I vs. NAC; *p*-Value ***
		All Patients (NAC and NAC+I); *n* = 22	Neoadjuvant Chemotherapy (NAC); *n* = 14	Neoadjuvant Chemotherapy and Pembrolizumab (NAC+I); *n* = 8	
Age *	Median Age	42.5 years	45 years	40 years	0.18 (WRS)
	Age Range	25–79 years	25–79 years	33–75 years	
Sex *	Female	22 (100%)	14 (100%)	8 (100%)	
Race **	White	11 (50.0%)	4 (28.6%)	7 (87.5%)	
	Black	9 (40.9%)	8 (57.1%)	1 (12.5%)	
	Native American	1 (4.6%)	1 (7.1%)	0	
	No Data	1 (4.6%)	1 (7.1%)	0	
Stage **	I	2 (9.1%)	1 (7.1%)	1 (12.5%)	
	II	6 (27.3%)	5 (35.7%)	1 (12.5%)	
	III	14 (63.6%)	8 (57.1%)	6 (75%)	
BMI (kg/m^2^) *	Average BMI	29.2	30	27.8	0.66 (WRS)
BMI Category **	<30 kg/m^2^	8 (36.4%)	8 (57.1%)	6 (75%)	
	≥30 kg/m^2^	14 (63.6%)	6 (42.9%)	2 (25%)	
Chemotherapy RDI *	Doxorubicin	82.2	91.5	67	
	Cyclophosphamide	81.8	89.7	69.1	
	Paclitaxel	94.3	93.6	95.4	
	Carboplatin ****	88.2	85.3	88.9	
Checkpoint Inhibitor Dose *	Pembrolizumab (number of cycles administered)	----	0	7.3	
Gene Expression Biomarkers *	Tumor immunogenic score (TIGS)	55.6	51.3	63	0.3 (WRS)
	Cell Proliferation (CP)	54.8	50.9	61.6	0.3 (WRS)
	Cancer Testis Antigen Burden (CTAB)	171	185.6	145.5	0.81 (WRS)
	PD-L1 (assessed by RNA-seq)	42.1	31	61.5	0.04 (WRS)
Response **	Pathological Complete Response (pCR)	16 (72.7%)	10 (71.4%)	6 (75.0%)	1 (FET)
	No Pathological Complete Response (Non-pCR)	6 (27.3%)	4 (28.6%)	2 (25.0%)	1 (FET)

* Continuous variables (average value in each category shown). ** Categorical variables (total number of patients in each category and corresponding percentage of entire cohort and each treatment group shown). *** *p*-values comparing NAC and NAC+I treatment groups. p-values only indicated if applicable. Statistical tests used to compute each *p*-value indicated (WRS = Wilcoxon Rank-Sum test, FET = Fisher’s exact test). **** Only two patients in the NAC group received carboplatin. BMI = body mass index; RDI = relative dose intensity.

**Table 2:** Genes significantly differentially expressed in pCR or non-pCR subgroups within each treatment group.

Treatment Group	Response Group	Upregulated Genes (FC > 2, *p* < 0.05)
NAC	pCR	***IL12B***, ***IL13***, *ADGRE5*
	Non-pCR	*MAPK14*, *IL1B*, *RB1*
NAC+I	pCR	***LCK****, **TP63***, *CEACAM1*, *HERC6*, *TCF7*, *CXCL1*, *CXCR5*, *ISG20*, *MX1*, *IFIT2*, *OAS3*, *IDO1*, *IFI44L*, *EIF2AK2*, *IKZF3*, *IL7R*
	Non-pCR	*PTPN11*, *CD63*, *ITGB1*, *LRP1*, *NRP1*, *FOXO1*, *GUSB*, *IKZF4*, *LAMP1*, *TNFSF4*, *MADCAM1*, *NOTCH3*, *EGR2*, *AXL*

Significantly differentially expressed genes exhibiting a fold change of greater than 10 are shown in bold.

## Data Availability

The datasets generated and/or analyzed during the current study are not publicly available due to a non-provisional patent filing covering the methods used to analyze such datasets, they but are available from the corresponding author upon reasonable request.

## Abbreviations

FDA	Food and Drug Administration
TNBC	Triple-negative breast cancer
irAEs	immune-related adverse events
TME	tumor microenvironment
pCR	pathological complete response
RNA	ribonucleic acid
FFPE	formalin-fixed paraffin-embedded
NAC	neoadjuvant chemotherapy administered without combination immunotherapy
NAC+I	neoadjuvant chemotherapy administered in combination with pembrolizumab immunotherapy
CPI	immune checkpoint inhibitor
U.S. FDA	United States Food and Drug Administration
DNA	deoxyribonucleic acid
CP	cell proliferation signature
TIGS	tumor immunogenic signature
CTAB	cancer testis antigen burden
CTA	cancer testis antigen
RDI	relative dose intensity
TILs	tumor infiltrating lymphocytes
OR	odds ratio
FC	fold change
